# Erectile dysfunction in hypertensive males in Kenya: a tertiary referral hospital experience

**DOI:** 10.4314/ahs.v22i2.48

**Published:** 2022-06

**Authors:** Malcolm C Correia, Elijah N Ogola, Joshua K Kayima, Mark D Joshi, David M Silverstein, Samuel K Kabinga

**Affiliations:** 1 University of Nairobi, College of Health Sciences, East African Kidney Institute, Nairobi, Kenya; 2 University of Nairobi, College of Health Sciences, Department of Clinical Medicine and Therapeutics, Nairobi Kenya; 3 The Nairobi Hospital, Nairobi, Kenya

**Keywords:** Erectile dysfunction, hypertensive males, Kenya

## Abstract

Erectile dysfunction (ED) in males can be organic or/and psychological.

**Objective:**

To determine the burden of ED among adult hypertensive men attending ambulatory clinic at Kenyatta National Hospital, Nairobi, Kenya.

**Methods:**

Descriptive cross-sectional study on patients on antihypertensive medications, followed for ≥ one month. The tools used were five-item International Index of Erectile Function (IIEF-5) for ED and Alcohol Use Disorders Identification Test for alcohol use.

**Results:**

Among 385 patients, the mean age was 56.2 ±11.3 years, median follow up in medical clinic was 5 years. The mean body mass index was 26.3 ± 4.6 kg/m2, 209(54.3%) were pre-obese/obese. Current smokers were 76(19.7%) while 133(34.5%) were former smokers. Alcohol use in the past year was reported by 256(68.5%), while hazardous alcohol intake was in 54(14%). Prescribed antihypertensives in various combinations included angiotensin converting enzyme inhibitors/angiotensin receptor blockers to 292(75.8%), calcium channel blockers to 238(61.8%), beta blockerso 129(33.5%) and thiazide diuretics on 77(20%). Using IIEF-5 tool, the prevalence of ED was 364(94.5%) (95%CI 92.2–96.6) distributed as mild in 255(70%), moderate 76(21.9%) and 33(9.1%) severe.

**Conclusion:**

The burden of ED is high among hypertensive males. Precipitants of ED like cigarette smoking and alcohol use and use of antihypertensives which can cause ED are prevalent too.

## Introduction

Erectile dysfunction (ED) is the inability to attain or maintain a penile erection sufficient for satisfactory vaginal intercourse.^1^ It has the phenotype of partial or total inability to consistently achieve and or maintain an erection. 2, 3 Erectile dysfunction as a disorder dates back to more than 5000 years among the Egyptian men.^4^, ^5^ Erectile dysfunction is a marker of cardiovascular disease.^6^, ^7^ It has been associated with coronary arterial disease (CAD), stroke, and all-cause mortality and may serve as a harbinger for other cardiovascular diseases in hypertensive population.^8^–^11^

The causes of ED span from organic to psychogenic sources in varied combinations.^2^, ^3^ They can be psychogenic, neurogenic, endocrinological, vasculogenic, drug-induced, dysfunction due to ageing, lifestyle factors and systemic diseases.^2^ The International Consultation Committee for Sexual Medicine on Definitions/Epidemiology/Risk Factors for Sexual Dysfunction reported prevalence rates between 10% to more than 90% among men aged 40 to 70 years with prevalence rates rising with advancement in age.^3^, ^12^–^15^

The prevalence of ED in hypertensive populations is higher, and has been reported to be more than double in men with hypertension when compared with age-matched normotensive men after controlling for other factors.^16^ The association between ED and hypertension spans from shared aetiology of age, cigarette smoking among others to common pathobiology encompassing endothelial dysfunction and atherosclerosis. A five item International Index of Erectile Function (IIEF-5) questionnaire is a psychometric tool developed to diagnose the presence and severity of erectile dysfunction (ED) and possesses favorable properties for detecting the presence and severity of ED with 98% sensitivity and 88% specificity.^17^ The tool consists of 5 domains assessing 15 items on erectile function and orgasmic function and are considered to reflect the physical findings and sexual desire, intercourse satisfaction, and overall satisfaction which reflect the psychological factors.^18^

The IIEF-5 assesses five domains. These include confidence in getting and keeping an erection, the frequency of erections hard enough for penetration, the frequency of maintaining erection after penetration, whether there are difficulties in maintaining an erection to completion of intercourse and whether the sexual intercourse is satisfactory to the individual. The tool provides scoring of patients with the lowest score being 5 and the highest 25. The higher the score, the less the severity of ED. A score of above 21 indicates no ED and a score of 11 or less indicates moderate to severe ED.

Standardized Alcohol Use Disorders Identification Test (AUDIT-C) questionnaire is a tool to capture alcohol use. The AUDIT-C classifies alcohol use by frequency of intake over the last 3 months into hazardous or non-hazardous alcohol use with a score above 4 for men being significant.

In Kenya, ED is presumed to be common though most patients do not consult the medically trained doctors. They prefer to consult the traditional medical practitioners because of the stigma and sociocultural taboos associated with erectile dysfunction.19 Patients with comorbidities like hypertension are likely to have even a higher prevalence of ED. It is in the background that this study aimed at establishing the burden of ED among the adult hypertensive males attending ambulatory clinic at Kenyatta National Hospital Nairobi Kenya (KNH). It was registered with Kenyatta National Hospital-University of Nairobi Ethics and Research committee number P632/11/2017.

## Materials and methods

### Design and setting

This was a descriptive cross-sectional study among ambulatory adult male patients on follow up for hypertension at KNH, a teaching and referral hospital in Kenyan capital city, Nairobi. It was carried out between January and July 2018.

### Study subjects

Male patients with a documented diagnosis of hypertension on antihypertensive medications and had been on followed up for at least one month were included. Those excluded were patients on gonadotrophin deprivation therapy, congestive heart failure stages 3 and 4, chronic kidney disease (CKD) stages 4 and 5, diabetic patients (self-reported and documented), or those who required hospital admission at the time of the interview.

### Sample size

The sample size of 384 was calculated from a single group using the Fisher's formula with a 5% margin of error. Convenient sampling was done at the medical out-patient clinics screening all males prior to the start of the clinic for eligibility and informed consent given.

### Study procedure

After enrolment, age, marital status, education level and employment status and the duration of hypertension from diagnosis were documented. Medical recorded were perused for documentation of chronic illnesses such as dyslipidemia, heart disease, angina, ischaemia and stroke which were collaborated from the history. Patients were further interviewed for history of cigarette smoking which was noted as current if presently smoking or quit within the last 5 years, former smoker if quit more than 5 years ago or never smoked. History of alcohol consumption was captured using the standardized Alcohol Use Disorders Identification Test (AUDIT-C) questionnaire. The clinical assessment using standard procedures for height and weight, were done and body mass index (BMI) calculated. The IIEF-5 was then administered.

### Data analyses

International Business Machine Statistical Package for Social Sciences (IBM-SPSS®) version 22 was used for analyses. Continuous variables presented as means with standard deviations if normally distributed or medians an interquartile ranges if otherwise. Categorical variables had frequencies and percentages calculated. Comparison of proportions for categorical variables by using the χ2 test and student t-test for normally distributed continuous variables. Logistic regression analyses were carried out with ED as the dependent variable and cigarette smoking, hazardous alcohol use and comorbidities like dyslipidemia, heart disease, angina, ischemia and stroke as covariates. A p-value below 0.05 was considered to be statistically significant.

## Results

**Figure 1:**
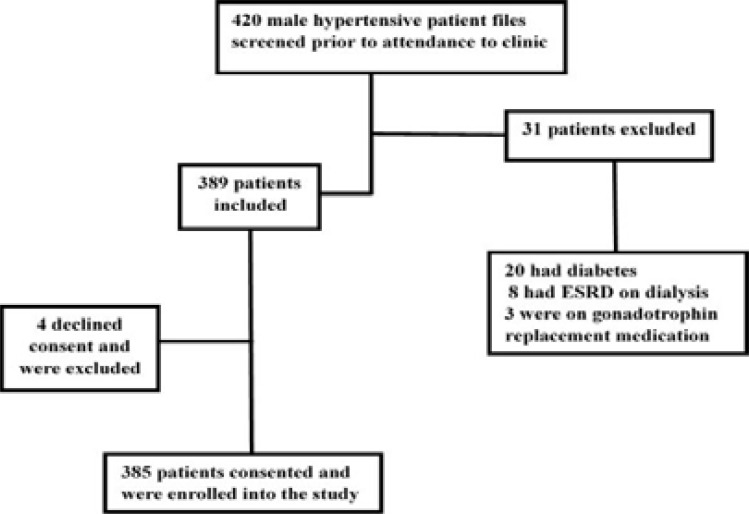
Patients recruitment flowchart

Out of 420 male patients screened, 35 were excluded and 385 were recruited into the study (Figure 1). The mean age was 56.2 ±11.3 years. About 355(92.2%) were married and 373(97%) had attained formal education. The median duration from diagnosis of hypertension was 5 years (Interquartile Range 3.0–10.0). A history of a first degree family member having hypertension was reported in a third of participants. Chronic illnesses reported were dyslipidaemia, heart disease, angina, heart attack, ischaemia and stroke. Angioplasty was reported by 8 patients. The mean body mass index (BMI) was 26.3 ± 4.6 kg/m^2^ and 209(54.3%) were pre-obese or obese. Current smokers were 76(19.7%) while 133(34.5%) were former smokers. Alcohol use in the past year was reported by 256(68.5%), while hazardous alcohol intake was reported in 54(14%) of respondents. Prescribed antihypertensives in various combinations included angiotensin converting enzyme inhibitors (ACEi) or angiotensin receptor blockers (ARB) to 292(75.8%), calcium channel blockers (CCB) to 238(61.8%), beta blockers at 129(33.5%) and 77(20%) were on thiazide diuretics. Other medication prescribed included aspirin (18.7%) and statins (16.9%). (Table 1). The scoring by IIEF-5 revealed marked difficulty in maintenance of erection to completion of intercourse and profound unsatisfactory coitus. (Table 2). The prevalence of ED was 94.5%(95% CI 92.2–96.6) The distribution of ED was mild (12–21 IIEF-5 score) in 255(70.1%), moderate (8–11 score) in 76(21.9%) and severe (5–7 score) in 33(9.1%). (Table 3).

**Table 1:** Characteristics of the respondents

Description/characteristic	All (n=385)	Those with severe ED (IIEF-5 (5–7)) n=33	Those with no ED (IIEF-5 (>21)) n=21
	Statistic	Statistic	Statistic
*Age±SD (year)*	56.3±11.3	58±12.9 p<0.001 (95% CI 53.36–62.54)†	52.0±4.5 p<0.001 (95% CI49.93–54.06)†
*Mean body mass index (kg/m^2^)*	26.3 ± 4.6	27.10±4.34 p<0.001(95%CI 25.51–28.59)†	25.35±1.35 p<0.001 (95% CI 24.68–25.91)†
*Underweight (<18.5) n(%)*	14(3.6)	0(0.00)	0(0.0)
*Normal (18.5–24.9) n(%)*	162(42.1)	12(36.4)	11(51.3)
*Pre-obesity (25.0–29.9) n(%)*	125(32.5)	10(30.3)	10(48.7)
*Obesity class I (30.0–34.9)* *n(%)*	68(17.6)	7(21.2)	0(0.0)
*Obesity class II (35.0 –* *39.9) n(%)*	16(4.2)	4(12.1)	0(0.0)
*Obesity class III (>40.0) n(%)*	0 (0.0)	0(0.00)	0(0.0)
*Median duration of follow up in* *hypertension clinic (year)*	5.0	11.0 p<0.001(95% CI 9.96–20.43)†	3.0 p<0.001 (95% CI 4.20–10.18)†
*Marital status*		p<0.001‡	p=0.007‡
*Married n(%)*	355(92.2)	29(87.9)	17(81.0)
*Single n(%)*	18(4.7)	0(00.0)	4(19.0)
*Divorced n(%)*	12 (3.1)	4(12.1)	0(0.0)
*Education*		p=0.486	p=0.156‡
*No formal education n(%)*	13(3.4)	0(0.00)	0(0.0)
*Primary n(%)*	93 (24.2)	19(57.6)	6(28.6)
*Secondary n(%)*	165(42.9)	14(42.4)	11(52.4)
*Tertiary n(%)*	114(29.6)	0(0.00)	4(19.0)
*Cigarette smoking*		p=0.035	p=0.156‡
*Current smoker n(%)*	76 (19.7)	18(54.5)	4(19.0)
*Former smoker n(%)*	133(34.5)	8(24.2)	6(28.6)
*Never smoked n(%)*	172(44.7)	7(21.2)	11(52.4)
*Alcohol use*		p<0.001	p=0.007‡
*Alcohol use in lifetime n(%)*	312(81)	29(87.9)	17(81.0)
*Used Alcohol in the past 1 year* *n(%)*	256(68.5)	29(87.9)	7(33.3)
*Hazardous alcohol use n(%)*	54(14)	8(24.2)	4(19.0)
*Family history of hypertension* *n(%)*	129(35.1)	p=0.728‡ 18(54.5)	p=0.078‡ 6(28.6)
*Co-morbidities*		p<0.001‡	p=1.00‡
*Dyslipidemia n(%)*	77(20.0)	19(57.6)	3(14.3)
*Heart disease n(%)*	22(5.7)	4(12.1)	0(0.0)
*Angina n(%)*	64(16.6)	4(12.1)	0(0.0)
*Heart attack n(%)*	28(7.3)	4(12.1)	0(0.0)
*Angioplasty n(%)*	8(2.1)	0(0.00)	0(0.0)
*Stroke n(%)*	37(9.6)	4(12.1)	0(0.0)
*Ischaemic n (%)*	8(2.1)	0(0.00)	0(0.0)
*Medications*			
*Alpha blockers n(%)*	18(4.7)	4(12.1)	0(0.0)
*Beta blockers n(%)*	129(33.5)	4(12.1)	14(66.7)
*ACEi n(%)*	118(30.6)	4(12.1)	0(0.0)
*ARB n(%)*	174(45.2)	26(78.8)	9(42.9)
*Calcium channel blocker n(%)*	238(61.8)	22(66.7)	3(14.3)
*Thiazide diuretics n(%)*	77(20.0)	0(0.00)	3(14.3)
*Non-thiazide diuretics n(%)*	53(13.8)	10(30.3)	4(19.0)
*Statins n(%)*	320(83.1)	7(21.2)	3(14.3)
*Aspirin n(%)*	313(81.3)	11(33.3)	7(33.3)

SD standard deviation, ACEi angiotensin converting enzyme inhibitor, ARB angiotensin receptor blocker, CI Confidence Interval

† t-test

‡ X^2^-test

**Table 2:** The 5-item International Index of Erectile Dysfunction

IIEF-5 entries	Interpretation (point)				
	Very Low (1)	Low (2)	Moderate (3)	High (4)	Very high (5)
How do you rate your confidence that you can get and keep your erection? n(%)	37(9.6)	172(44.7)	139(36.1)	23(6.0)	14(3.6)
	Never or almost never	A few times	Sometimes	Most times	Almost always or always
When you had erections with sexual stimulation, how often were your erections hard enough for penetration? n(%)	46(11.9)	122(31.7)	119(30.9)	77(20.0)	21(5.5)
During sexual intercourse how often were you able to maintain your erection after you had penetrated (entered) your partner? n(%)	42(10.9)	127(33.0)	98(25.5)	98(25.5)	20(5.2)
During sexual intercourse, how difficult was it to maintain your erection to completion of intercourse? n(%)	36(9.4)	133(34.5)	60(15.6)	112(29.1)	44(11.4)
When you attempted sexual intercourse, how often was it satisfactory for you? n(%)	73(19.0)	136(35.3)	57(14.8)	91(23.6)	28(7.3)

IIEF-5; 5-item International Index of Erectile Dysfunction

**Table 3:** Erectile Dysfunction Score

Severity of Erectile Dysfunction by IIEF-5 score (score)	Number n(%)
No Erectile Dysfunction (22–25)	21(5.5)
Mild Erectile Dysfunction (12–21)	255(66.2)
Moderate Erectile Dysfunction (8–11)	76(19.7)
Severe Erectile Dysfunction (5–7)	33(8.6)
**Total**	**385(100.0)**

This study did not find association between ED and age below or above 50 years. The duration of hypertension from diagnosis did not have a strong association with ED. Any history of smoking had an association with increased ED with an OR 1.3 although it was not statistically significant (p > 0.528). Hazardous alcohol consumption had a trend toward a protective association OR 0.7 but it was not statistically significant (p > 0.515). (Table 4) Hazardous alcohol use, smoking status (ever smoked or never smoked), positive history of heart disease, angina, ischemia, dyslipidemia and stroke predicted the presence of ED by 93.8% accuracy (Table 5).

**Table 4:** Association between erectile dysfunction and age below or above 50 years, median duration of hypertension, smoking and alcohol use

Variable n=364	Erectile dysfunction n (%)	No erectile dysfunction n (%)	OR (95% CI)	P value
**Age in years**				
**<50**	111 (96.5)	4 (3.5)	1.0	
**50+**	253 (93.7)	17 (6.3)	0.5 (0.2–1.6)	0.265
**Median duration of HTN** **(IQR) in years**	5.0 (3.0–10.0)	3.0 (3.0–15.0)	-	0.957
**Ever smoked**				
**Yes**	199 (95.2)	10 (4.8)	1.3 (0.6–3.2)	0.528
**No**	165 (93.8)	11 (6.2)	1.0	
**Alcohol consumption in** **past 1 year**	50 (92.6)	4 (7.4)	0.7 (0.2–2.1)	0.515
**Hazardous**	314 (94.9)	17 (5.1)	1.0	
**Nonhazardous**				

OR odds ratio, CI confidence interval, n number

**Table 5:** Prediction of erectile dysfunction from presence of hazardous alcohol use, smoking, heart disease, angina, ischaemia, dyslipidemia and stroke

Observed		Predicted		
		Erectile Dysfunction		Percentage Correct
		absent	present	
Erectile Dysfunction	absent	0	6	0.0
	present	0	91	100.0
Overall Percentage				93.8

## Discussion

One essential measure of a good quality of life is an active and healthy sexual life. Sexual health and cardiovascular disease share common risk factors which include arterial hypertension, dyslipidemia, obesity, and smoking. These were prevalent in this study population. The common mediating mechanisms are endothelial dysfunction, subclinical inflammation, and atherosclerosis. Hypertension burden increases with age and ED is 2–3 times more in men with hypertension.^20^ Advancement in age, vascular system wellbeing and erectile function are interlinked.^21^ Erectile dysfunction has been noted to precede vascular diseases.^22^, ^23^ It has prevalence rates of about 10% in males aged 40 years, and 4 in every 5 among men over 70 years of age. It is higher among those with coronary artery disease (CAD), than in those without. 6, 24

A five item International Index of Erectile Function (IIEF-5) has been used to diagnose the presence and severity of ED and possesses favorable properties for detecting the presence and severity.(17) In this study, the prevalence of ED was very high. Majority had mild ED, with almost 1 in 10 men presenting with severe ED. Those with severe ED were older, higher BMI and longer duration of follow up in hypertension clinic. More were active cigarette smokers when compared with those who had no ED. The IIEF-5 tool has been used elsewhere. In Spain, patients with systemic arterial hypertension evaluated using IIEF-5 had more than 50% of the studied men had severe ED, and there was an independent association between ED, age, years ofdiagnosis of hypertension and number of treatments^25^, findings concordant to those of this study. In China, among male with hypertension and ischaemic stroke, the incidence of ED as was almost 80% and there was no significant difference in the age, smoking, alcohol consumption and BMI between the ED group and the non-ED group when evaluated by IIEF-5.^26^ This differed from our findings probably due to higher selection of the respondents.

Despite the study population being younger, ED was more prevalent which might points to more factors than age being at play. Majority of patients were on ACE inhibitors, ARBs and calcium channel blockers (CCB). Antihypertensive medications have been known to affect erectile function negatively with exception of short-acting angiotensin receptor blockers.^27^ This coupled with the median long duration of hypertension may account for the high prevalence of ED showed in the study. Other factors like, depression and anxiety which increase ED were not evaluated in this study. If ED precedes CVD, majority of the patients in this study are candidates for evaluation for CAD among others.

## Limitations

This study doES not favour generalizability to the general population include the hospital-based, single centre tertiary level health facility which could have selected for patients with more severe or advanced disease being enrolled into the study.

## Conclusion

The burden of ED among hypertensive males is high. Known factors which can aggravate ED like cigarette smoking and alcohol use are also prevalent among this population. The choice of antihypertensive medications in these male patients might also have played a role in increasing the burden of ED. Screening for ED, in hypertensive male patients, by use IIEF-5 questionnaire if routinely used may lead to earlier diagnosis, adjustment of antihypertensive medications and advice on lifestyle changes including cigarette smoking cessation.
